# The effect of passive ultrasonic activation of 2% chlorhexidine or 3% sodium hypochlorite in canal wall cleaning

**DOI:** 10.4317/jced.52088

**Published:** 2015-02-01

**Authors:** Carmen Llena, Carla Cuesta, Leopoldo Forner, Sandra Mozo, Juan-Jose Segura

**Affiliations:** 1 MD,DDS,PhD, Department of Stomatology. Universitat de València, Spain; 2 DDS, Private practice; 3 MD,DDS,PhD, Department of Stomatology. Universidad de Sevilla, Spain

## Abstract

Objectives: the purpose of this study was to compare debris removal and open tubules effectiveness of sodium hypochlorite (NaOCl) and chlorhexidine (CHX) applied as final irrigation in different protocols Study Design: sixty extracted premolars were divided into six groups according to the final irrigation technique: A and B 3 % NaOCl or 2 % CHX with the Miraject needle and no agitation; C and D, passive ultrasonic irrigation (PUI) with Irrisafe 20 tips and 3 % NaOCl or 2 % CHX; E and F, PUI with Irrisafe 25 tips and 3 % NaOCl or 2% CHX. The remaining dentine debris and opened tubules were evaluated by SEM at three root levels by two blinded investigators. The Kruskal Wallis and the Mann-Whitney U test was used to compare groups and levels, with a significance of p<0.05. Results: Debris elimination was significantly higher in PUI groups (p<0.05). PUI groups showed a higher capability to open tubules, compared to groups A and B. In the coronal third, groups D to F eliminated more debris and opened more tubules than conventional irrigation (p<0.05). In medium third, group E eliminated significantly more debris (1.60) than group A (2.60). No differences were obtained among groups in apical third. Both NaOCl and CHX applied with PUI showed no differences in debris elimination or opened tubules. Conclusions: Final PUI with Irrisafe tips was the most effective procedure for eliminating the debris and opening up dentinal tubules, independent of the irrigant solution or Irrisafe type size.

** Key words:**Ultrasonic irrigation, PUI, sodium hypochlorite, chlorhexidine.

## Introduction

Irrigating solutions act as desinfectant, lubricant and cleaning agent during biomechanical preparation, improving the elimination of the contaminated dentin and the permeability of the canal (1). Recommended irrigation protocols include sodium hypoclorite (NaOCl), alone or combined with ethylenediaminetetraacetic acid (EDTA) or chlorhexidine (CHX) (2). As safety and effective irrigation sequence it has been proposed the initial use of NaOCl during instrumentation, followed by EDTA and a final irrigation with CHX (3).

It has been shown that irrigating solutions can only progress 1 mm beyond the tip of the needle (4), and that the network of side canals cannot be cleaned mechanically. Cleaning improves moving the irrigating solution into the canal (5). So, different ultrasonic techniques have been proposed to improve irrigant distribution (6), such as irrigation combined with simultaneous ultrasonic instrumentation (UI) and passive ultrasonic irrigation (PUI) (7). When PUI is used, energy is transmitted from a file or smooth oscillating wire to the irrigant by ultrasonic waves, producing a stream and cavitation of the irrigating solution (8,9). PUI reduces the potential to create deformities and can be used with a continuous or intermittent irrigant flow (10), both techniques have proved to be equally effective in removing dentin residues from root canal when used for three minutes (11).

The use of NaOCI combined with ultrasound or a wave vibration system is the irrigation method with the greatest antibacterial effect (12). Nevertheless, Weber *et al.* (13) evidenced prolonged antimicrobial action of CHX vs. NaOCl when applied with ultrasonic activation.

The aim of this study was to compare the ability of two final irrigating solutions, NaOCl 3% or CHX 2%, using three irrigation techniques (conventional syringe and intermittent PUI with Irrisafe 20 and 25 tips) to remove the debris and open dentin tubules at three canal wall levels. The null hypotheses are: a) the irrigation technique with conventional syringe is equally efficient in eliminating debris and open tubules as final rinsing techniques using intermittent PUI; b) there are no differences in canal wall cleaning using NaOCl or CHX as final irrigation.

## Material and Methods

The Ethical Committee of the University of Valencia (Spain) approved this study. Sixty extracted single-rooted human premolars, were used for this study and placed in 3% NaOCl solution for 5 minutes and stored in saline solution. Each root had one canal, with a curvature less than 5 degrees under X-ray inspection on 3 angulated films. Teeth were coronally sectioned to obtain a working length of 16 mm. A size 10 K-file (Dentsply Maillefer, Ballaigues, Switzerland) was passively introduced into the root canal until its tip was visible at the apical foramen. Subtracting 1 mm from this length the working length (WL) was established. All the canals were shaped by the same operator with the MTwo rotary system (VDW, Munich, Germany) basic sequence (10/.04, 15/.05, 20/.06, 25/.06) and a 30/.05 rotary file. Irrigation with 2.5 mL of 3% NaOCI (Ultradent Products, South Jordan, UT) was used between files, with a 27G Miraject needle (Hager Werken, Duisburg-Grobenbaum, Germany), with a round ended lateral exit inserted 1mm shorter than the working length. The total volume of NaOCl used during instrumentation was 12.5 ml. After instrumentation, teeth were rinsed for 2 minutes with 2 ml 10% EDTA (Tubuliclean; OGNA LAB, Muggiò, Italy) followed by a 3-minute final rinse with 2 ml of saline solution. Then canals were dried with paper points.

**Figure 1:**
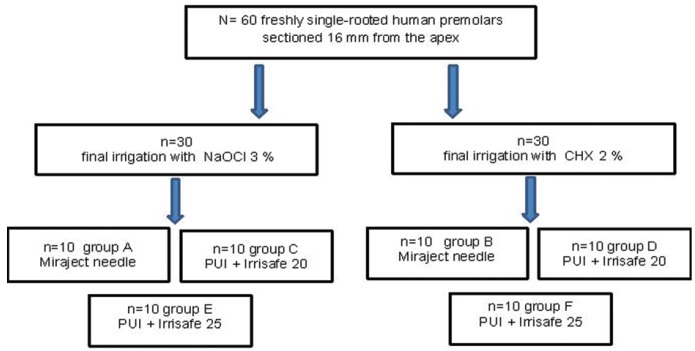
Groups distribution.

Teeth were randomly divided into six groups (n = 10) according to the final irrigation technique (Fig. 1), as follows: groups A and B, irrigation with 3% NaOCl or 2 % CHX, respectively, applied with the Miraject needle (Hager Werken, Duisburg-Grobenbaum, Germany), with a round ended lateral exit inserted 1mm shorter than the working length, and no ultrasonic agitation; groups C and D, 3% NaOCl or 2 % CHX, respectively, using PUI with Irrisafe 20 tips (Acteon, Merignac, France); groups E and F, irrigation with 3% NaOCl or 2% CHX, respectively, using PUI with Irrisafe #20/.00 and #25/.00 (Acteon, Merignac, France) tips. activated through a 5.5 W 30 kHz piezoelectric ultrasound Suprasson P5 Booster unit (Satelec Acteon, Merignac, France). PUI was conducted in groups C to F, with intermittent flush (3 cycles x 20 sec of ultrasonic activation). Refrigeration between cycles was performed with 2 mL of NaOCI or CHX (depending on the experimental group). Ultrasonic tip was placed 1 mm coronal to the working length, the file was kept centered in the canal, and 2-3 mm apical-coronal movements were made (5-9).

-SEM analysis

After canal preparation, lines were marked on the root surfaces, dividing them into thirds at 2, 6, and 10 mm from the apical aspect. Then, teeth were sectioned along their bucco-lingual surfaces as follow, two longitudinal and symmetrical grooves were performed in the external root surface with a low speed diamond bur (Isomet, Buehler, Lake Bluff, NY, USA), then with a hand chisel two halves were obtained. The root canal half with the most visible part of apex was selected. Sectioned roots were dried and mounted on metallic stubs, sputter-coated with a gold-palladium alloy (Polaron Range SC 7620, Watfora, UK) and evaluated at the apical, middle and coronal levels under a field emission SEM (Geol JSM-6060VL, USA). In order to standardize the area to be examined, the central beam of the SEM was directed to the center of each third of the root canal under 30X magnification which was increased to 1000X. The selected area was captured on the screen of the SEM to score it. One image per third was taken and coded. All observations were carried out by two blinded investigators. Three images by group were randomly selected and evaluated after 2 months. Inter- and intra-observer agreements were carried out by Cohen´s Kappa test.

**Figure 2:**
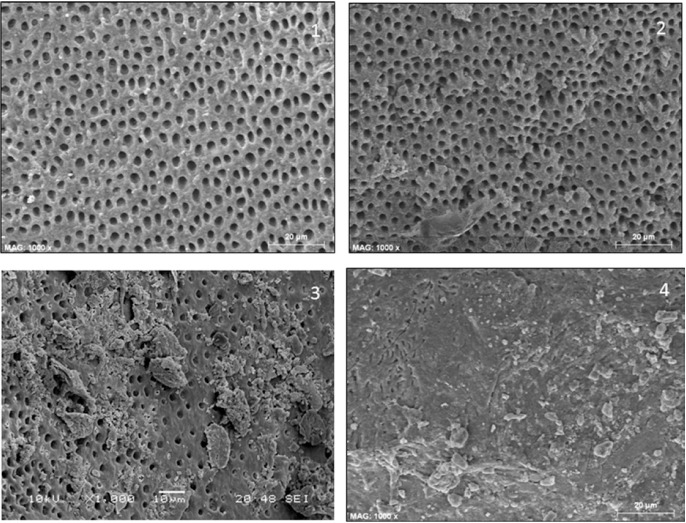
Amount of debrir. 1: no debris; 2: <50% of surface covered by debris; 3: 50-75% of surface covered by debris; 4: more than 75% of surface covered by debris.

**Figure 3:**
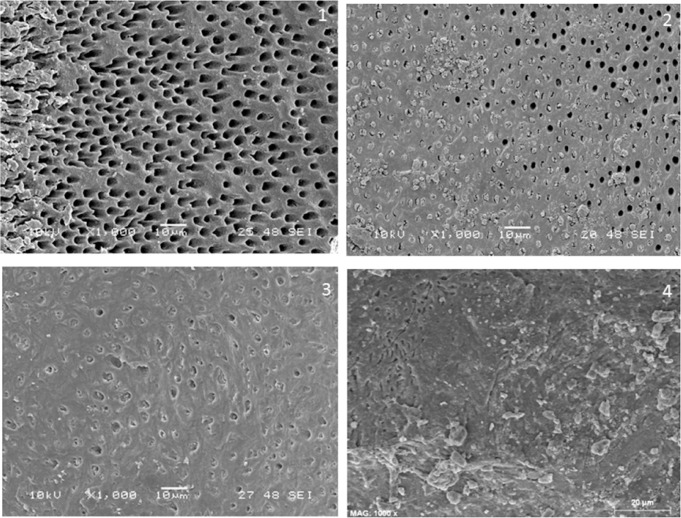
Amount of opened tubules. 1: all opened; 2: 50-75% of opened tubules; 3: <50% of opened tubules; 4: all dentin tubules were closed.

The amount of debris was marked from 1 to 4 (modified Hülsmann & Stoz ) (14): 1, no debris; 2, <50% of surface covered by debris; 3, 50-75% of surface covered by debris; 4, more than 75% of surface covered by debris (Fig. 2). Amount of opened dentinal tubules was evaluated according to the following criteria: 1, all opened; 2, 50-75% of opened tubules; 3, <50% of opened tubules; 4, all dentin tubules were closed (Fig. 3).

Data were analysed with SPSS 19.0 (SPSS Inc., Chicago, USA) statistical software using the Kruskal Wallis and the Mann-Whitney U test. Statistically significant differences of the data was set at *p* < 0.05.

## Results

Inter-examiner agreement was 0.89 and the intra-examiner 0.91 and 0.86 respectively (for both examiners).

As it can be seen in table 1, debris elimination was significantly higher in PUI groups (*p*<0.05), but no differences were found between irrigating solutions or Irrisafe type size. In the coronal third, groups D, E and F eliminates more debris than group A (conventional irrigation with NaOCL) (*p*<0.05) ( Table 1). In the middle third, only PUI + Irrisafe 25 + NaOCl, (group E) eliminates significantly more debris than conventional irrigation with NaOCl ( Table 1). There are no significant differences between groups in the apical third (*p*>0.05), but PUI groups showed less debris than conventional ones ( Table 1).

**Table 1:**
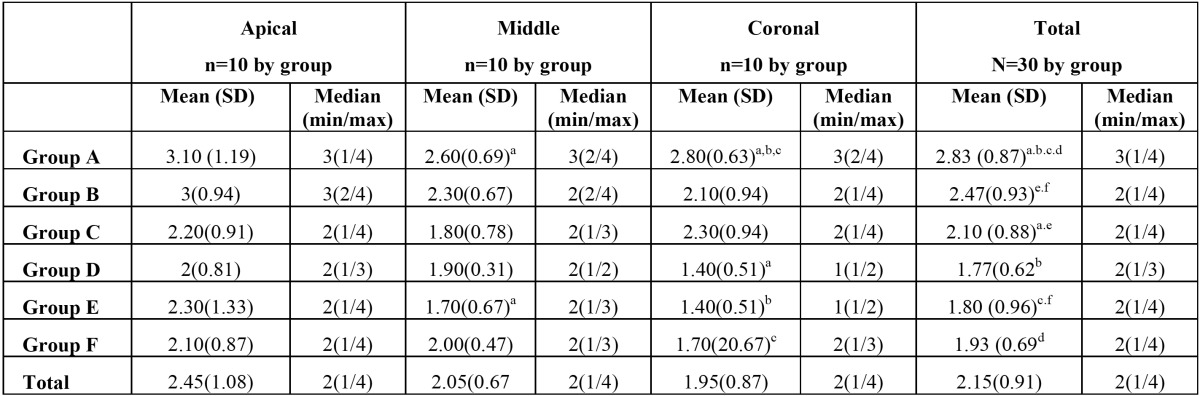
Mean, standar deviation, median, minimum and maximun of debris by group.

PUI groups (C to F) showed a higher capability to open tubules compared to groups A and B, but significant differences were only observed with conventional NaOCl irrigating solution (group A). In the coronal third groups D, E and F shows more dentinal opened tubules than group A ( Table 2). We did not find significant differences among groups (*p*>0.05) in the middle and apical third ( Table 2).

**Table 2:**
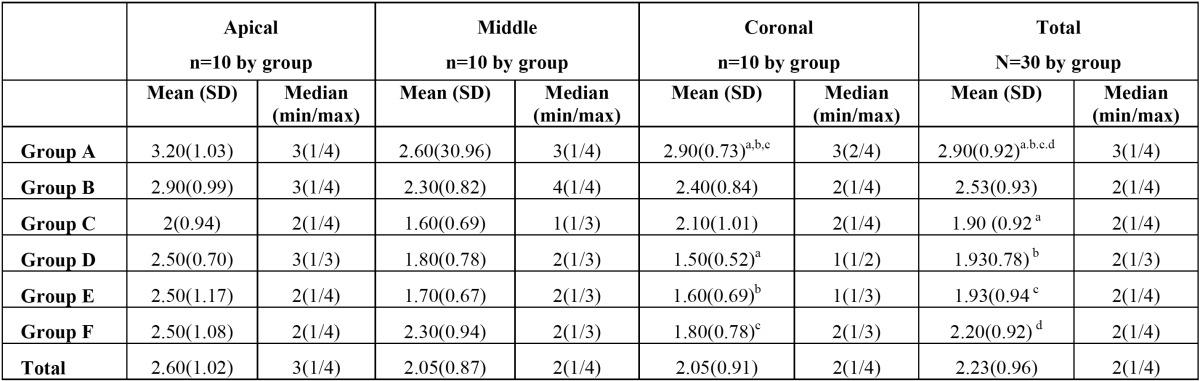
Mean, standar deviation, median, minimum and maximun open tubules by group.

There were no significant differences (*p*>0.05) among thirds in any group for debris or opened tubules.

## Discussion

Use of SEM has recently been questioned due to the limitations of two-dimensional images (8), but the ideal experimental model to assess smear layer removal is not currently available (15). Paqué *et al.* (16) described a µCT method that may provide three-dimensional data but not at the SEM level. Several procedures have been used to evaluate irrigants and techniques efficiency: perforation simulations and artificial placed dentine debris (6,11) or simulated lateral canals associated to simulated root perforations (17). Nevertheless, many authors follow the methodology used in the present study (5,18-21).

Findings in our study reject the null hypothesis that the conventional syringe is as efficient than intermittent PUI in eliminating debris and open tubules. But not reject that there are no differences in debris elimination or opened tubules using NaOCl or CHX as final irrigation.

PUI with Irrisafe tips and an intermittent flush technique of three cycles of 20 second applications is just as effective in eliminating the debris as laser activated techniques, however one single 20 second application with Irrisafe tips and ultrasound was significantly less effective (22). Time which irrigant remains in the canal is a factor to be taken into account during PUI, with a longer period there is higher risk of contact between instruments and canal walls, with the consequent injuries production inside the canals (23). In our study 3 cycles of ultrasonic activation for 20 seconds were performed with files inserted centred in the canal.

Instrument must be able to move freely in the canal during ultrasound irrigation as contact of the instrument with the walls would limit the flush of irrigation throughout the canal system, reducing the effectiveness of cleaning and disinfection (1). In this study, no effectiveness differences were found in debris removal or exposure of tubules in relation to tip size in the PUI groups.

It is stated that the best moment to activate the irrigant is after canal instrumentation, so that we can introduce the ultrasonic tip along the working length, which increases the irrigation efficacy (8,24).

Despite any of the described techniques it is possible to eliminate smear layer completely or, at least, to eliminate all organic debris (4,25), there is a general consensus that PUI is more effective than conventional syringe and needle irrigation at eliminating debris (1,21,26), which coincides with the findings of this study. PUI eliminates more dentin debris than conventional irrigation at all evaluated root-levels, but only with significant differences in apical and middle thirds, independent of the final irrigant (NaOCl or CHX).

The majority of the related studies evaluated NaOCl action as final irrigant (2,22,27). We have found two papers only where CHX (as final irrigant) capability to clean smear layer and to open tubules have been analyzed: Ferreira *et al.* (25), found CHX to be less effective than NaOCl or an mixed ultrafiltered (CHX and NaOCl) ultrasonic applied in a continuous way; nevertheless, Vasconcelos *et al.* (28) did not find differences in smear layer cleaning an opening tubules efficiency between NaOCl and CHX used as final irrigants after EDTA application.

Some studies analyzed CHX as final PUI-applied irrigant related to its antimicrobial action (29-32), suggesting effectiveness for a longer time because of it substantivity.

Results obtained with CHX with regard to debris elimination and amount of opened dentinal tubules in addition to substantivity, offer an interesting alternative. The following sequence could be proposed: NaOCl irrigation during instrumentation, followed by EDTA and final PUI-activated irrigation with CHX.

In this in vitro experiment, the majority of the remaining debris was located in the apical third. The same is reported by the great majority of authors (5,33,34). But no significant differences were obtained among the evaluated thirds in any group, this findings are in accordance with Castagna *et al.* (21).

## Conclusions

It can be concluded that irrigation with conventional syringe in the initial preparation stage, followed by 10% EDTA and a final phase of passive ultrasound irrigation (PUI) with intermittent flush and Irrisafe tips, is effective for cleaning root canals, independently of the use of CHX or NaOCl as final irrigant.
